# *DOCK7-ANGPTL3* SNPs and their haplotypes with serum lipid levels and the risk of coronary artery disease and ischemic stroke

**DOI:** 10.1186/s12944-018-0677-9

**Published:** 2018-02-17

**Authors:** Wei-Jun Li, Rui-Xing Yin, Xiao-Li Cao, Wu-Xian Chen, Feng Huang, Jin-Zhen Wu

**Affiliations:** 1 Department of Cardiology, Institute of Cardiovascular Diseases, The First Affiliated Hospital, Guangxi Medical University, 22 Shuangyong Road, Nanning, 530021 Guangxi People’s Republic of China; 2 Department of Neurology, The First Affiliated Hospital, Guangxi Medical University, Nanning, 530021 Guangxi People’s Republic of China

**Keywords:** Dedicator of cytokinesis 7, Angiopoietin like 3, Single nucleotide polymorphisms, Lipid, Coronary artery disease, Ischemic stroke

## Abstract

**Background:**

Little is known about the association of the dedicator of cytokinesis 7 (*DOCK7* rs1748195) and angiopoietin like 3 (*ANGPTL3* rs12563308) single nucleotide polymorphisms (SNPs) and their haplotypes with serum lipid levels and the risk of coronary artery disease (CAD) and ischemic stroke (IS) in the Chinese populations. This study aimed to detect such association in a Southern Chinese Han population.

**Methods:**

This study included 1728 subjects (CAD, 568; IS, 539; and controls, 621). Genotypes of the two SNPs were determined by the Snapshot technology.

**Results:**

The genotypic and allelic frequencies of the rs1748195 SNP were different between CAD patients and controls (*P* < 0.05 for each), the rs1748195G allele frequency was higher in CAD patients than in controls (27.6% vs. 23.6%, *P* = 0.024). The genotypic frequencies of the rs12563308 SNP were also different between CAD patients and controls (*P* = 0.021). The rs1748195 SNP was associated with an increased risk of CAD after controlling for potential confounders and Bonferroni correction (*P* < 0.025 considered statistically significant; Recessive: OR = 1.79, 95% CI = 1.04-3.06, *P* = 0.017; Log-additive: OR = 1.27, 95% CI = 1.02-1.57, *P* = 0.014), whereas the rs12563308 SNP was associated with a decreased risk of CAD (Dominant: OR = 0.69, 95% CI = 0.45-0.94, *P* = 0.011; Log-additive: OR = 0.73, 95% CI = 0.49-0.89, *P* = 0.009). The rs1748195 SNP was also associated with an increased risk of severity to coronary artery atherosclerosis (Dominant: OR = 1.45, 95% CI = 1.07-2.11, *P* = 0.017; Log-additive: OR = 1.35, 95% CI = 1.09-1.82, *P* = 0.013). The interactions of SNP-environment on serum lipid levels and the risk of severity to coronary artery atherosclerosis, CAD and IS were noted. The rs1748195G-rs12563308T haplotype was associated with an increased angiographic severity to coronary artery atherosclerosis (OR = 1.46, 95% CI = 1.05-2.03), and the risk of CAD (OR = 1.37, 95% CI = 1.08-1.74). The interactions of haplotype-hypertension on the risk of CAD and haplotype-drinking on the risk of CAD/IS were observed.

**Conclusions:**

These results suggest that the *DOCK-ANGPTL3* SNPs and their haplotypes were associated with the angiographic severity to coronary artery atherosclerosis and the risk of CAD and IS in the Southern Chinese Han population.

## Background

The mortality and morbidity of coronary artery disease (CAD) and ischemic stroke (IS) remain very high in different districts [1–3]. The Global Burden of Disease (GBD) 2015 study reported that the global number of deaths due to cardiovascular disease had ranged from 12.59 million in 1990 to 17.92 million in 2015. Meanwhile, the global number of deaths due to IS in 2015 was 24.93 million [4]. The prevention and treatment of the two diseases remain a great challenge to us. Though both diseases are complex multifactorial disorder, it is widely accepted that the pathological basis of the two diseases are atherosclerosis, and serum dyslipidemia plays an important role in process of coronary artery atherosclerosis [5]. The view that genetic and environmental factors such as sex, age, obesity, cigarette smoking, alcohol consumption, hypertension and hyperlipidemia contribute to suffering both the diseases was confirmed by different researches [6–12] and powerful guidelines have token the related factors into considerations when coming to prevention of the diseases [8, 9]. Previous genome-wide association studies (GWASes) have identified many genetic variants influenced the risk of CAD or IS [13].

The dedicator of cytokinesis 7 gene (*DOCK7*) is located in chromosome 1p31. It encodes a guanine nucleotide exchange factor (GEF) that plays a role in axon formation and neuronal polarization. Angiopoietin like 3 gene (*ANGPTL3*) encodes a member of a family of secret proteins that function in angiogenesis. The encoded protein, which is expressed predominantly in the liver, is further processed into an N-terminal coiled-coil domain-containing chain and a C-terminal fibrinogen chain. The N-terminal chain is important for lipid metabolism, while the C-terminal chain may be involved in angiogenesis (http://www.ncbi.nlm.nih.gov/gene/). The protein was proven to increase serum cholesterol and triglyceride (TG) levels in mice [14] and in humans [15]. *ANGPTL3* lies within an intron of *DOCK7*, and previous GWASes showed that single nucleotide polymorphisms (SNPs) in the *DOCK7-ANGPTL3* were associated with serum lipid traits, not only total cholesterol (TC) but also TG [16, 17]. Recently, Graham et al. [18] reported that ANGPTL3 retarded the progression of atherosclerosis and reduced atherogenic lipoprotein levels in oligonucleotides targeting mice. Furthermore, using the same strategy to target human ANGPTL3, they also found that the ANGPTL3 could reduce atherogenic lipoprotein levels in humans including serum TG, LDL-C, very low density lipoprotein (VLDL) cholesterol, non-high density lipoprotein cholesterol (non-HDL-C), apolipoprotein (Apo) B, and ApoCIII levels [18]. In several previous studies, we have investigated the association of the *DOCK7* rs1168013, *DOCK7* rs10889332 SNPs with serum lipid levels [19], and *DOCK7* rs10889353, *DOCK7* rs10889335 with serum lipid levels and the risk of CAD and IS [20]. In a recent study in a Chinese pediatric population, Shen et al. [21] have showed that the *DOCK7* rs1748195 SNP was strongly associated with TG and HDL-C. However, the association of the *DOCK7* rs1748195 and *NGPTL3* rs12563308 SNPs with the risk of CAD and IS has not been detected previously. Therefore, the present study aimed to explore such association in a Southern Chinese Han population.

## Methods

### Patients

A total of 1107 unrelated participants were recruited from hospitalized patients in the First Affiliated Hospital, Guangxi Medical University. There were 568 CAD and 539 IS patients. CAD group included stable angina and acute coronary syndrome (unstable angina, non-ST segment elevation myocardial infarction and ST segment elevation myocardial infarction). The diagnosis of CAD based on typical angina or discomfort, electrocardigraphic changes (ST-segment depression or elevation of ≥0.5 mm, T-wave inversion of ≥ 3 mm in ≥ 3 leads, or left bundle branch block), increases in the cardiac makers (creatinine kinase-MB and troponin T or I), as well as positive coronary angiograms (coronary stenosis ≥ 50% in at least one of the three main coronary arteries or their major branches which reviewed by two independent angiographers who were both blinded to the clinical and genotype results) [22]. The angiographic severity of coronary artery atherosclerosis was classified according to the number of coronary vessels with significant stenosis (luminal narrowing ≥ 50%) as one-, two-, or three-vessel disease in the three major coronary arteries. Some CAD patients had taken medicines before they were admitted to hospital. A total of 171 (30.11%) patients used one or more than one lipid-lowing drugs such as fibrates or statins. All of the IS patients received a strict neurological examination and brain magnetic resonance imaging (MRI). The diagnosis and classification of IS was according to the Trial of Org 10,712 in Acute Stroke Treatment (TOAST) criteria, and patients included met one or two criteria: large-artery thrombosis and small-vessel occlusion [23]. There were 149 patients (27.64%) using lipid-lowing drugs. The participants with a history of autoimmune, hematologic, neoplastic, liver, renal, thyroid, and type 1 diabetes were rejected. CAD subjects with a history of IS or IS participants with a history of CAD were not included in this study. There were 58 patients not included in this study because of the co-existence of both CAD and IS.

### Controls

A total of 621 control subjects matched by age, gender, ethnic group (Han nationality in Guangxi, China) were included. All of the individuals were randomly selected from the Physical Examination Center of the First Affiliated Hospital during the same period. Questionnaires, history-taking, strict clinical examination and image examinations (computed tomography or MRI) were used to insure all the participants free of CAD and IS. They did not take drugs known to affect serum lipid levels (such as statins or fibrates, betablockers, diuretics, or hormones). All participants have provided their written informed consents and the study protocol was approved by the Ethics Committee of the First Affiliated Hospital, Guangxi Medical University (No. Lunshen 2009-Guike-018; Jan. 7, 2009). The reported investigations were in accordance with the principles of the Declaration of Helsinki.

### Biochemical measurements

A venous blood sample of 5 ml was obtained from all participants after at least 12 h of fasting. A part of the sample (2 ml) was collected into glass tubes and used to measure serum lipid levels, another part (3 ml) was stored in the tubes contained anticoagulants (4.80 g/L citric acid, 14.70 g/L glucose, and 13.20 g/L tri-sodium citrate) and used to extract deoxyribonucleic acid (DNA). The interval between the disease attack and blood collection for analysis was within 36 h. The levels of serum TC, TG, HDL-C, and LDL-C were determined by enzymatic methods with commercially available kits (RANDOX Laboratories). Serum ApoA1 and ApoB levels were detected by the immunoturbidimetric immunoassay. The normal values in our Clinical Science Experiment Center were 3.10-5.17 mmol/L for TC, 0.56–1.70 mmol/L for TG, 0.91–1.81 mmol/L for HDL-C, 2.70–3.20 mmol/L for LDL-C, 1.00–1.78 g/L for ApoA1, 0.63–1.14 g/L for ApoB, and 1.00–2.50 for the ApoA1/ApoB ratio. The participants with TC > 5.17 mmol/L, and/or TG > 1.70 mmol/L were defined as hyperlipidemic [24, 25]. Hypertension was defined as a systolic blood pressure of 140 mmHg or greater, and/or a diastolic blood pressure of 90 mmHg or higher [26]. Drinking based on alcohol consumption (yes or no). Individuals’ age was divided into > 60-year or ≤ 60-year subgroups. Body mass index (BMI) was calculated according to the values of weight divided by height squared (kg/m^2^). A BMI of ≤ 24, 24-28, and > 28 kg/m^2^ was defined as normal weight, overweight and obesity; respectively. Smoking was defined as current smoking (yes or no).

### SNP selection

The selection of SNPs was according to the following assumption: (1) Selected SNPs were Tagging SNPs established by Haploview (Broad Institute or MIT and Harvard, Cambridge, MA, USA, version 4.2); (2) SNP information was obtained from NCBI dbSNP Build 132 (http://www.ncbi.nlm. nih.gov/SNP/); (3) SNPs were restricted to the minor allele frequency (MAF) > 1%; and (4) SNPs in the gene might be associated with the serum lipid levels or cardiovascular disease in previous studies [14–21].

### Genotyping

Genomic DNA was extracted from leucocytes of venous blood using the phenol-chloroform method. Genotyping of the SNPs was accomplished by the Snapshot technology platform in the Center for Human Genetics Research, Shanghai Genesky Bio-Tech Co. Ltd. [25, 27–29]. The restriction enzymes for the loci were SAP (Promega) and Exonucleasel (Epicentre). The sense and antisense primers were 5’-AGAGGAGGAGCTCCATTCTTATATTTTTG-3′ and 5’-AACACTGCAAGTCTGTCTCACATAGGA-3′ for the rs1748195 SNP; and 5’-GGAGAATTTTGGTTGGGCCTAGA-3′ and 5’-TGCTTTGTGATCCCAAGTAGAAAACA-3′ for the rs12563308 SNP; respectively.

### Statistical analyses

The statistical software of SPSS22.0 (SPSS Inc., Chicago, IL, USA) was used to carry out the statistical analyses. Quantiative variables were expressed as mean ± standard deviation (serum TG levels were expressed as medians and interquartile ranges). Qualitative variables were expressed as percentage. Allele frequency was determined via direct counting, and the standard goodness-of-fit test was used to test the Handy-Weinberg equilibrium (HWE). A chi-square analysis was used to evaluate the genotype distribution. The student’s unpaired *t*-test was used to test the general characteristics between patient and control groups. Analysis of covariance (ANCOVA) was used to test the association of genotypes and serum lipid parameters. The interactions of SNP- or haplotype-environment on serum lipid levels were detected by factorial regression analysis after controlling for potential confounders. Bonferroni correction was employed for variants associated with serum lipid levels, and a *P-*value < 0.025 was considered statistical significant (corresponding to *P* < 0.05 after adjusting for two independent tests). Meanwhile, the *P-*value of interaction (*P*_I_) ≤ 0.005 was considered statistically significant after Bonferroni correction. The association of genotypes and angiographic severity to atherosclerosis was detected by the unconditional logistic regression after adjusting related environmental factors, and the gene-environment interactions on the angiographic severity to atherosclerosis were tested by polytomous logistic regression for ordinal response. The association of genotypes and the risk of CAD and IS, also the SNP- or haplotype-environment interactions on the risk of CAD and IS were tested by the unconditional logistic regression after gender, age, BMI, smoking, alcohol consumption, hypertension and hyperlipidemia were adjusted [8, 9, 20, 25]. The correlation risk was estimated by odds ratio (OR) and 95% confidence interval (95% CI) and *P* < 0.025 considered statistical significant after Bonferroni correction. The pattern of pair-wise linkage disequiblirium (LD) between the two SNPs was measured by *D’* and *r*^2^ using the SHEsis software [30]. A two-tailed *P-*value less than 0.05 was considered statistically significant for the remaining variables. Haplotype frequency was determined by means of the algorithms implemented in the PHASE program.

## Results

### General characteristics of the participants

As shown in Table 1, the mean values of BMI, systolic blood pressure, pulse pressure, and ApoB levels were higher but diastolic blood pressure, alcohol consumption, TC, HDL-C and LDL-C levels were lower in CAD patients than in controls (*P* < 0.05 for all). The values of BMI, systolic blood pressure, diastolic blood pressure, pulse pressure levels were higher but alcohol consumption, TC, HDL-C and LDL-C levels were lower in IS patients than in controls.

**Table 1:** General characteristic of the participants

Characteristic	Control (*n* = 621)	CAD (*n* = 568)	IS (*n* = 539)	*P*_CAD_	*P*_IS_
Male/Female	449/172	418/150	389/150	0.617	0.960
Age, year	61.67 ± 11.99	62.26 ± 10.55	62.81 ± 12.37	0.739	0.868
BMI (kg/m^2^)	22.65 ± 3.20	23.91 ± 3.26	23.39 ± 3.54	0.006	0.009
Systolic blood pressure, mmHg	128.26 ± 19.00	133.16 ± 23.49	147.52 ± 22.00	0.000	0.004
Diastolic blood pressure, mmHg	80.56 ± 11.41	79.19 ± 14.27	83.71 ± 12.93	0.022	0.003
Pulse pressure, mmHg	47.70 ± 13.74	53.98 ± 17.68	63.81 ± 17.76	0.000	0.000
Cigarette smoking, n (%)	244 (39.2)	244 (43.0)	221 (41.6)	0.199	0.553
Alcohol consumption, n (%)	270 (43.4)	130 (22.9)	150 (28.2)	0.000	0.000
Total cholesterol, mmol/L	4.88 ± 1.06	4.54 ± 1.20	4.52 ± 1.15	0.007	0.031
Triglyceride, mmol/L	1.37 (1.78)	1.67 (1.12)	1.62 (1.38)	0.748	0.465
HDL-C (mmol/L)	1.90 ± 0.48	1.14 ± 0.34	1.23 ± 0.40	0.000	0.000
LDL-C (mmol/L)	2.73 ± 0.78	2.71 ± 1.00	2.68 ± 0.90	0.000	0.000
Apolipoprotein (Apo) A1, g/L	1.41 ± 0.27	1.02 ± 0.32	1.02 ± 0.23	0.895	0.051
ApoB, g/L	0.90 ± 0.21	0.91 ± 0.27	0.89 ± 0.24	0.000	0.080
ApoA1/ApoB	1.64 ± 0.51	1.30 ± 1.83	1.27 ± 0.60	0.210	0.745

*CAD* coronary artery disease, *IS* ischemic stroke, *HDL-C* high-density lipoprotein cholesterol, *LDL-C* low-density lipoprotein cholesterol, *P*_CAD_, the *P* value between CAD patients and controls; *P*_IS_, the *P* value between IS patients and controls. The value of triglyceride was median (interquartile range), the difference between CAD/IS patients and controls was determined by the Wilcoxon-Mann-Whitney test

### Genotypic and allelic frequencies of the subjects

The genotypic and allelic frequencies of the *DOCK7-ANGPTL3* polymorphisms are summarized in Table 2. The genotype distribution of the two SNPs was consistent with the Hardy-Weinberg equilibrium in CAD/IS patients and controls (*P* > 0.05 for all). For the *DOCK7* rs1748195 SNP, the genotypic and allelic frequencies were different between CAD patients and controls (*P* < 0.05 for each), the frequency of the rs1748195G allele was higher in CAD patients than in controls (27.6% vs. 23.6%, *P* = 0.024). For the *ANGPTL3* rs12563308 SNP, the genotypic frequencies were also different between CAD patients and controls (*P* = 0.021). But no significant difference in the genotypic and allelic frequencies of the two SNPs was observed between IS patients and controls.

**Table 2:** Genotypic and allelic frequencies of the *DOCK7-ANGPTL3* SNPs in controls and cases

SNP/Genotype/Allele	Control (%)	CAD (%)	IS (%)	*P*_CAD_	*P*_IS_
Rs1748195					
CC	361 (58.1)	303 (53.3)	311 (57.7)		
CG	227 (36.6)	216 (38.0)	188 (34.9)		
GG	33 (5.3)	49 (8.7)	40 (7.4)	0.047	0.321
C	949 (76.4)	822 (72.4)	810 (75.1)		
G	293 (23.6)	314 (27.6)	268 (24.9)	0.024	0.476
*P*_HWE_	0.728	0.240	0.123		
CC/CG	588 (94.7)	519 (91.3)	499 (92.6)		
GG	33 (5.3)	49 (8.7)	40 (7.4)	0.024	0.140
Rs12563308					
TT	534 (86.0)	517 (91.0)	479 (88.8)		
CT	84 (13.5)	48 (8.5)	57 (10.6)		
CC	3 (0.5)	3 (0.5)	3 (0.6)	0.021	0.305
T	1152 (92.8)	1082 (95.2)	1015 (94.2)		
C	80 (7.2)	54 (4.8)	63 (5.8)	0.067	0.518
*P*_HWE_	0.876	0.112	0.364		
TT	534 (86.0)	517 (91.0)	479 (88.8)		
CT/CC	87 (14.0)	51 (9.0)	60 (11.2)	0.007	0.142

*SNP* single nucleotide polymorphisms, *CAD* coronary artery disease, *IS* ischemic disease, *HWE* Hardy- Weinberg equilibrium, *P*_HWE_ the *P* value of the Hardy-Weinberg equilibrium, *P*_CAD_ the *P* value between CAD patients and controls, *P*_IS_ the *P* value between IS patients and controls

### Genotypes and serum lipid levels

As shown in Table 3, we found that the ***DOCK7*** rs1748195 and ***ANGPTL3*** rs12563308 SNPs were not associated with serum lipid traits in the controls (*P* > 0.025 for all).

**Table 3:** Association of the *DOCK7-ANGPTL3* SNPs and serum lipid levels in controls

Genotype	n	TC (mmol/L)	TG (mmol/L)	HDL-C (mmol/L)	LDL-C (mmol/L)	ApoA1 (g/L)	ApoB (g/L)	ApoA1/ApoB
Rs1748195								
CC	361	4.92 ± 1.11	1.37 (0.90)	1.91 ± 0.47	2.70 ± 0.77	1.42 ± 0.28	0.89 ± 0.20	1.66 ± 0.53
CG	227	4.83 ± 1.00	1.18 (0.70)	1.88 ± 0.49	2.74 ± 0.80	1.40 ± 0.27	0.91 ± 0.22	1.62 ± 0.51
GG	33	4.98 ± 0.74	1.25 (0.69)	1.93 ± 0.55	2.85 ± 0.68	1.40 ± 0.20	0.94 ± 0.21	1.56 ± 0.40
*F*	–	0.661	2.381	0.327	0.685	0.434	0.959	0.893
*P*	–	0.517	0.063	0.721	0.504	0.648	0.384	0.410
CC + CG	588	4.88 ± 1.07	1.32 (0.84)	1.90 ± 0.48	2.72 ± 0.78	1.41 ± 0.28	0.90 ± 0.21	1.65 ± 0.52
GG	33	4.98 ± 0.74	1.19 (0.69)	1.94 ± 0.55	2.85 ± 0.68	1.40 ± 0.20	0.94 ± 0.21	1.56 ± 0.40
*F*	–	0.283	0.171	0.172	0.994	0.108	0.933	0.931
*P*	–	0.595	0.679	0.678	0.318	0.742	0.334	0.335
Rs12563308								
TT	534	4.90 ± 1.08	1.42 (0.89)	1.91 ± 0.49	2.73 ± 0.79	1.42 ± 0.28	0.90 ± 0.21	1.64 ± 0.48
CT	84	4.80 ± 0.87	1.21 (0.87)	1.86 ± 0.46	2.68 ± 0.72	1.38 ± 0.21	0.89 ± 0.20	1.66 ± 0.70
CC	3	5.41 ± 0.83	1.17 (0.47)	1.99 ± 0.50	3.11 ± 1.05	1.43 ± 0.11	1.08 ± 0.26	1.39 ± 0.43
*F*	–	0.842	0.466	0.450	0.553	0.826	1.260	0.409
*P*	–	0.431	0.627	0.638	0.575	0.438	0.284	0.665
TT	534	4.90 ± 1.09	1.42 (0.89)	1.91 ± 0.49	2.73 ± 0.79	1.42 ± 0.28	0.90 ± 0.21	1.64 ± 0.48
CT + CC	87	4.80 ± 0.87	1.20 (0.86)	1.86 ± 0.46	2.69 ± 0.71	1.38 ± 0.20	0.89 ± 0.21	1.65 ± 0.69
*F*	–	0.672	0.993	0.671	0.195	1.566	0.108	0.014
*P*	–	0.413	0.334	0.413	0.659	0.211	0.742	0.906

*TC* total cholesterol, *TG* triglyceride, *HDL-C* high-density lipoprotein cholesterol, *LDL-C* low-density lipoprotein cholesterol, *ApoA1* apolipoprotein A1, *ApoB* apolipoprotein B. The value of triglyceride was presented as median (interquartile range), and the difference among the different genetic models was determined by Kruskal-Wallis test. A *P* < 0.025 was considered statistically significant after Bonferroni correction

### *DOCK7-ANGPTL3* SNPs and the risk of CAD and IS

The associations of *DOCK7-ANGPTL3* SNPs and the risk of CAD and IS are shown in Table 4. The SNPs of rs1748195 and rs12563308 were associated with the risk of CAD after controlling for potential confounders including gender, age, BMI, cigarette smoking, alcohol consumption, hypertension and hyperlipidemia (a *P* < 0.025 was considered statistically significant after Bonferroni correction). The rs1748195 SNP was associated with an increased risk of CAD (Recessive: OR = 1.79, 95% CI = 1.04-3.06, *P* = 0.017; Log-additive: OR = 1.27, 95% CI = 1.02-1.57, *P* = 0.014), whereas the rs12563308 SNP was associated with a decreased risk of CAD (Dominant: OR = 0.69, 95% CI = 0.45-0.94, *P* = 0.011; Log-additive: OR = 0.73, 95% CI = 0.49-0.89, *P* = 0.009). However, no significant association was found between the two SNPs and IS.

**Table 4:** Genotypes of the two *DOCK7-ANGPTL3* SNPs and the risk of CAD and IS

SNP/Model	Ref. Genotype	Effect Genotype	(OR 95% CI)_CAD_	*P*_CAD_	IS (OR 95% CI)_IS_	*P*_IS_
rs1748195						
Codominant	CC	CG	1.16 (0.88-1.54)	0.058	0.93 (0.69–1.6)	0.221
		GG	1.90 (1.10–3.29)		1.59 (0.89–2.84)	
Dominant	CC	CG/GG	1.25 (0.96–1.64)	0.098	1.01 (0.76–1.35)	0.933
Recessive	CC/CG	GG	1.79 (1.04–3.06)	0.017	1.63 (0.92–2.88)	0.092
Overdomiant	CC/GG	CG	1.09 (0.83–1.43)	0.552	0.89 (0.66–1.19)	0.439
Log–additve	–	–	1.27 (1.02–1.57)	0.014	1.09 (0.87–1.37)	0.450
Rs12563308						
Codominant	TT	CT	0.67 (0.44–1.03)	0.192	0.75 (0.48–1.16)	0.408
		CC	1.17 (0.17–7.99)		1.28 (0.15–11.62)	
Dominant	TT	CT/CC	0.69 (0.45–0.94)	0.011	0.76 (0.49–1.17)	0.206
Recessive	TT/CT	CC	1.23 (0.18–8.44)	0.834	1.33 (0.15–11.62)	0.799
Overdomiant	TT/CC	CT	0.67 (0.44–1.03)	0.068	0.75 (0.48–1.16)	0.187
Log–additve	–	–	0.73 (0.49–0.89)	0.009	0.79 (0.53–1.19)	0.264

*SNP* single nucleotide polymorphisms, *CAD* coronary artery disease, *IS* ischemic disease, *OR (95%CI)* Odds ratio (OR) and 95% confidence interval (CI) between patients and controls

### *DOCK7-ANGPTL3* SNPs and the angiographic severity of CAD

As shown in Table 5, the rs1748195 SNP was associated with an increased risk of severity to coronary artery atherosclerosis measured by coronary angiography in CAD patients in different genetic models (Dominant: OR = 1.45, 95% CI = 1.07-2.11, *P* = 0.017; Log-additive: OR = 1.35, 95% CI = 1.09-1.82, *P* = 0.013). There was no association of the rs12563308 SNP and the risk of severity to coronary artery atherosclerosis in our research.

**Table 5:** Effect of the *DOCK7–ANGPTL3* SNPs on angiographic severity of CAD

SNP/Genotype	Rs1748195			Rs12563308		
		OR (95% CI)	*P*		OR (95% CI)	*P*
Codominant	CC	1		TT	1	
	CG	1.40 (0.94–2.08)	0.142	TC	0.89 (0.46–1.70)	0.859
	GG	1.71 (0.81–3.61)		CC	0.57 (0.05–6.73)	
Dominant	CC	1		TT	1	
	CG/GG	1.45 (1.07–2.11)	0.017	CT/CC	0.86 (0.46–1.63)	0.660
Recessive	CC/CG	1		TT/CT	1	
	GG	1.49 (0.72–3.10)	0.271	CC	0.58 (0.05–6.80)	0.671
Log–additve	C	1		T	1	
	G	1.35 (1.09–1.82)	0.013	C	0.86 (0.48–1.54)	0.614

Based to different number of main vessel stenosis by coronary angiography in CAD patients, Polytomous Logistic Regression for Ordinal Response was employeed to assess the severity, and environmental factors like sex, age, BMI, cigarette smoking, alcohol consumption, hypertension, hyperlipidemia were adjusted. A *P* < 0.025 was considered statistically significant after Bonferroni correction

### SNP-environment interactions on serum lipid levels and coronary atherosclerosis

**Fig. 1:**
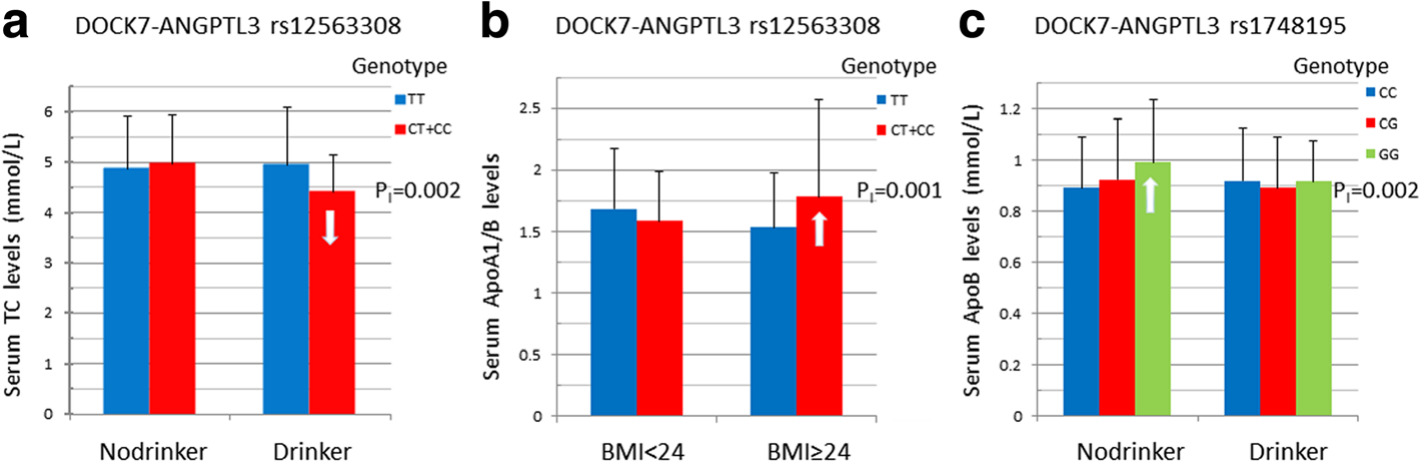
Interactions of the *DOCK7-ANGPTL3* SNPs and sex, age, BMI, smoking and drinking on serum lipid levels in controls. TC, total cholesterol; BMI, body mass index; ApoA1/ApoB, apolipoprotein A1/ apolipoprotein B ratio. The differences in serum TC levels and ApoA1/ApoB ratio among the genotypes were assessed using analysis of covariance. The interactions of the genotypes and alcohol consumption or BMI ≥ 24 kg/m^2^ on serum lipid levels were detected by using a factorial regression analysis after controlling for potential confounders (*P*_I_). **a**, the rs12563308CT/CC genotypes interacted with alcohol to decrease (↓) serum TC levels; **b**, the rs12563308CT/CC genotypes interacted with BMI ≥ 24 kg/m2 to increase(↑) the ApoA1/ApoB ratio; and c, the rs1748195GG genotype interacted with alcohol to decrease (↓) serum ApoB levels

The interactions of the *DOCK7-ANGPTL3* SNPs and sex, age, BMI, smoking, and drinking on serum lipid levels and the severity to atherosclerosis in CAD patients are shown in Table 6 and Fig. 1. The rs12563308 SNP interacted with alcohol consumption to decrease serum TC levels (Fig. 1a), and interacted with BMI ≥ 24 kg/m^2^ to increase the ApoA1/ApoB ratio (Fig. 1b). The rs1748195 SNP interacted with alcohol consumption to decrease serum ApoB levels (Fig. 1c). We also found that the rs1748195 SNP interacted with BMI to influence the severity to atherosclerosis in CAD patients. In CAD patients with BMI ≤ 24 kg/m^2^, the rs1748195G allele carriers had higher risk of severity to coronary artery atherosclerosis than the rs1748195G allele non-carriers (*P*_CAS_ = 0.02, OR = 1.83, 95% CI = 1.07-3.12).

**Table 6:** The *P*_I_ values for interactions of genotypes and drinking, smoking, and BMI on serum lipid levels and angiographic severity of coronary atherosclerosis in CAD

SNP/Factor	Lipid							*P*_CAS_
	TC	TG	HDL-C	LDL-C	ApoA1	ApoB	ApoA1/B	
Rs1748195								
Sex	0.987	0.417	0.561	0.942	0.403	0.632	0.948	0.18
Age	0.591	0.790	0.435	0.145	0.842	0.621	0.957	0.40
BMI	0.810	0.705	0.728	0.878	0.446	0.493	0.481	0.02
Smoking	0.552	0.876	0.840	0.194	0.209	0.816	0.949	0.50
Drinking	0.374	0.609	0.903	0.483	0.564	0.002	0.142	0.98
Rs12563308								
Sex	0.457	0.603	0.840	0.849	0.707	0.998	0.867	0.85
Age	0.800	0.982	0.140	0.247	0.104	0.365	0.210	0.56
BMI	0.380	0.517	0.209	0.236	0.307	0.082	0.001	0.17
Smoking	0.596	0.941	0.497	0.384	0.473	0.618	0.527	0.45
Drinking	0.002	0.562	0.180	0.267	0.099	0.228	0.208	0.31

*SNP* single nucleotide polymorphism, *TC* total cholesterol, *TG* triglyceride, *HDL-C* high-density lipoprotein cholesterol, *LDL-C* low-density lipoprotein cholesterol, *ApoA1* apolipoprotein A1, *ApoB* apolipoprotein B, *CAD* coronary artery disease, *IS* ischemic stroke, *BMI* body mass index. The *P*_I_ values for interactions were detected by ANCOA, and a *P* ≤ 0.005 was considered statistically significant after Bonferroni correction; *P*_CAS_, the *P* interaction value of angiographic severity to coronary atherosclerosis in CAD patients, and the Polytomous Logistic Regression for Ordinal Response was employeed. A *P*_CAS_ ≤ 0.025 was considered statistically significant after the rest four environmental factors was adjusted when one of the environmental factors interactions was detected

### SNP-environment interactions on the risk of CAD and IS

**Fig. 2:**
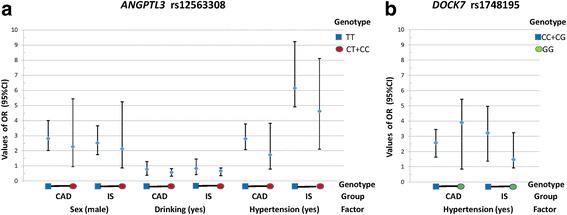
Gene-environment interactions on the risk of CAD and IS. **a** the rs12563308 SNP interacted with sex (male), drinking (yes) or hypertension (yes) to influence serum lipid levels in CAD and IS patients; **b** the rs1748195 SNP interacted with hypertension (yes) to influence serum lipid levels in CAD and IS patients; CAD, coronary artery disease; IS, ischemic stroke; OR (95% CI), the odds ratio (OR) and 95% confidence interval (95% CI)

As shown in Table 7 and Fig. 2, the rs12563308TT-sex (male) interaction increased the risk of CAD and IS (Fig. 2a; *P*_CAD_ = 0.016, OR = 2.82, 95% CI = 2.02-4.00; *P*_IS_ = 0.07, OR = 2.52, 95% CI = 1.74-3.66). But the rs12563308CT/CC-drinking (yes) interaction decreased the risk of CAD and IS (Fig. 2a; *P*_CAD_ = 0.010, OR = 0.57, 95% CI = 0.32-0.82; *P*_IS_ = 0.013, OR = 0.68, 95% CI = 0.35-0.87). The rs12563308TT-hypertension (yes) interaction increased the risk of CAD (Fig. 2a; *P*_CAD_ = 0.019, OR = 2.80, 95% CI = 2.08-3.78) but not IS (*P*_IS_ = 0.582). The rs1748195CC/CG-hypertension (yes) interaction increased the risk of CAD (Fig. 2b; *P*_CAD_ = 0.005, OR = 2.58, 95% CI = 1.63-3.45) but not IS (*P*_IS_ = 0.458).

**Table 7:** Gene-environment interactions on the risk of CAD and IS

Factor	Rs1748195		Rs12563308	
	*P*_CAD_	*P*_IS_	*P*_CAD_	*P*_IS_
Sex (male vs. female)	0.507	0.932	0.016	0.007
Age (≤ 60 vs. > 60 year)	0.972	0.374	0.482	0.411
BMI (≤24 vs. > 24 kg/m^2^)	0.741	0.299	0.389	0.135
Smoking (yes vs. no)	0.905	0.811	0.827	0.851
Drinking (yes vs. no)	0.983	0.902	0.010	0.013
Hypertension (yes vs. no)	0.005	0.458	0.019	0.582
Hyperlipidemia (yes vs. no)	0.744	0.980	0.453	0.123

*P*_CAD_, the *P* value between CAD patients and controls; *P*_IS_, the *P* value between IS patients and controls; *BMI* body mass index. The *P* values for interactions of genotypes and sex, age, BMI, drinking, smoking, hypertension, hyperlipidemia on the risk of CAD/IS were obtained from unconditional logistic regression after all the environmental factors were adjusted, and a *P* < 0.025 was considered statistically significant after Bonferroni correction

### LD analyses, haplotypes and the risk of atherosclerosis and CAD/IS

As shown in Table 8, strong LD was found between the two SNPs in CAD and IS patients (*D’* = 0.88–0.91). Haplotype analyses showed that the haplotype of rs1748195G-rs12563308T was associated with an increased angiographic severity to atherosclerosis (OR = 1.46, 95% CI = 1.05-2.03). The haplotype of rs1748195G-rs12563308T was also associated with an increased risk of CAD (OR = 1.37, 95% CI = 1.08-1.74) but not IS.

**Table 8:** Linkage disequilibrium analysis of the *DOCK7–ANGPTL3* SNPs and the association of haplotypes and the risk of atherosclerosis, CAD and IS

Group	Haplotype	Control Fre.	Case Fre.	OR (95% CI)	*P*	LD	
						*D’*	*r* ^2^
CAS	C–T	0.7643	0.7064	1			
	G–T	0.1815	0.2486	1.46 (1.05–2.03)	0.023	–	–
	G–C	0.0541	0.0434	0.93 (0.51–1.69)	0.820		
	Rare	*	*				
CAD	C–T	0.7554	0.7224	1			
	G–T	0.1722	0.2300	1.37 (1.08–1.74)	0.009	0.8838	0.3833
	G–C	0.0638	0.0464	0.90 (0.59–1.37)	0.630		
	Rare	*	*				
IS	C–T	0.7554	0.7514	1			
	G–T	0.1722	0.1902	1.12 (0.87–1.45)	0.372	0.9060	0.4263
	G–C	0.0638	0.0584	0.95 (0.62–1.46)	0.811		
	Rare	*	*				

*Control Fre.* the frequency of haplotypes in controls, *Case Fre.* the frequency of haplotypes in CAD/IS patients, *CAS* coronary atherosclerosis. Group CAS, according to angiographic severity in CAD patients, only one–vessel and more than one–vessel in the three major in the three major coronary arteries were defined as control and case respectively; Group CAD/IS, all the patients in our research; the risk of severity to atherosclerosis and CAD/IS was obtained by unconditional logistic regression, and a *P* < 0.05 was considered statistically significant after sex, age, BMI, drinking, smoking, hypertension, hyperlipidemia were adjusted; *, the values of the rare haplotypes were not listed

### Haplotype-environment interactions on the risk of CAD and IS

The interactions of several haplotypes and environment factors on the risk of CAD/IS were also noted in this study. When one of the environmental factors was detected, the rest six factors of sex, age, BMI, smoking, alcohol consumption, hypertension and hyperlipidemia were adjusted.

For the interactions of the haplotypes and hypertension on the risk of CAD/IS, as compared with the same haplotype in normotensive participants, the rs1748195C-rs12563308T-hypertension (OR = 2.98, 95% CI = 2.07-4.30) and rs1748195G-rs12563308T-hypertension (OR = 2.49, 95% CI = 1.66-3.74) interactions were associated with an increased risk of CAD. For the normotensive subjects, as compared with the rs1748195C-rs12563308T haplotype, the rs1748195G-rs12563308T haplotype (OR = 1.47, 95% CI = 1.09-1.99) was associated with an increased risk of CAD.

For the interactions of the haplotypes and drinking on the risk of CAD/IS, as compared with the same haplotype in non-drinkers, the rs1748195G-rs12563308T-drinking (OR = 0.71, 95% CI = 0.36-0.89) and rs1748195G-rs12563308C-drinking (OR = 0.26, 95% CI = 0.11-0.58) interactions were associated with a decreased risk of CAD. The rs1748195G-rs12563308T-drinking (OR = 0.38, 95% CI = 0.23-0.61) and rs1748195G-rs12563308C-drinking (OR = 0.24, 95% CI = 0.10-0.55) interactions were also associated with a decreased risk of IS. For the non-drinkers, as compared with the rs1748195C-rs12563308T haplotype, the rs1748195G-rs12563308T haplotype was associated with an increased risk of CAD (OR = 1.38, 95% CI = 1.04-1.84).

## Discussion

The results of the present study showed that the genotypic and allelic frequencies of the *DOCK7* rs1748195 SNP were different between CAD and controls (*P* < 0.05 for each), the G allele frequency was significantly higher in CAD than in controls (27.6% vs. 23.6%, *P* = 0.024). The genotypic frequency of the *ANGPTL3* rs12563308 SNP was also different between CAD and controls (*P* = 0.021). The *DOCK7* rs1748195 SNP and rs1748195G-rs12563308T haplotype were associated with an increased risk of CAD (Recessive: OR = 1.79, 95% CI = 1.04-3.06, *P* = 0.017, Log-additive: OR = 1.27, 95% CI = 1.02-1.57, *P* = 0.014; OR = 1.37, 95% CI = 1.08-1.74, *P* = 0.009; respectively) and the severity to coronary atherosclerosis (OR = 1.46, 95% CI = 1.05-2.03, *P* = 0.023), whereas the *ANGPTL3* rs12563308 SNP was associated with a decreased risk of CAD (Dominant: OR = 0.69, 95% CI = 0.45-0.94, *P* = 0.011; Log-additive: OR = 0.73, 95% CI = 0.49-0.89, *P* = 0.009). The interactions of rs12563308-drinking on decreased TC levels; rs12563308-BMI (≥ 24 kg/m^2^) on increased ApoA1/ApoB ratio; rs1748195-BMI (≤ 24 kg/m^2^) on increased the severity to coronary atherosclerosis; rs12563308TT-hypertension, rs1748195CC/CG-hypertension, rs1748195C-rs12563308T-hypertension and rs1748195G-rs12563308T-hypertension on increased risk of CAD; rs1748195G-rs12563308T-drinking and rs1748195G-rs12563308C-drinking on decreased risk of CAD; rs1748195G-rs12563308T-drinking and rs1748195G-rs12563308C-drinking on decreased risk of IS; rs12563308TT-sex (male) on increased risk of CAD and IS; and rs12563308CT/CC-drinking on decreased risk of CAD and IS were also observed. To our best knowledge, the present study is the first report to explore the effects of the two *DOCK7-ANGPTL3* SNPs, their haplotypes, SNP- or haplotype-environment interactions on coronary atherosclerosis and the risk of CAD and IS.

In the present study, we showed that the genotypic and allelic frequencies of the *DOCK7* rs1748195 SNP and the genotypic frequency of the *ANGPTL3* rs12563308 SNP were different between CAD and controls. According to the data from international HapMap project, the rs1748195C and rs1748195G allele frequencies were 74.3% and 25.7% in CHB (Han Chinese in Beijing, China); 65.4% and 34.6% in GBR (British in England and Scotland); 71.2% and 28.8% in MSL (Mende in Sierra Leone); and 59.7% and 40.3% in GWD (Gambian in western divisions in the Gambia). The frequencies of rs12563308T and rs12563308C alleles were 94.2% and 5.8% in CHB; 88.1% and 11.9% in GWD; and 84.1% and 15.9% in MSL. These results suggest that the prevalence of the *DOCK7* rs1748195 and *ANGPTL3* rs12563308 SNPs might have a racial/ethnic specificity. But more and larger samples of molecular epidemiological investigations are necessary to confirm our findings.

Previous GWASes reported that some *DOCK7-ANGPTL3* polymorphisms were associated with serum TG and TC levels [17, 31]. Shen et al. [20] showed that the rs1748195 SNP was strongly associated with TG and HDL-C in a Chinese pediatric population. In the present study, however, we found that the *DOCK7* rs1748195 and *ANGPTL3* rs12563308 SNPs were not associated with all of seven serum lipid traits in the controls (*P* > 0.025 for all). The reason for these discrepancies in different investigations is still unclear. In addition to different genetic background, sample size, different statistical method, and different gene-gene or gene-environment interactions may also contribute to the discrepancies among our study and other studies in different populations. Interestingly, in this study in controls, we found that the *ANGPTL3* rs12563308 SNP interacted with alcohol consumption to decrease serum TC levels, and interacted with BMI ≥ 24 kg/m^2^ to increase the ApoA1/ApoB ratio. The *DOCK7* rs1748195 SNP also interacted with alcohol consumption to decrease serum ApoB levels. These results suggest that the mechanism of the two SNPs to influence lipid metabolism may be different. For the *DOCK7* rs1748195 SNP, it not only affected promoter factors to bind motif Hoxa11*,* but also made effect on post-transcriptional regulation when its base sequence was changed [32]. But this phenomenon was not observed in the *ANGPTL3* rs12563308 SNP. We speculated that incomplete linkage-disequilibrium of the *DOCK7* rs1748195 SNP and carried different haplotypes with other SNPs may change genes’ functions. Thus, the two *DOCK7-ANGPTL3* SNPs may be indirectly involved in lipid metabolism.

**Fig. 3:**
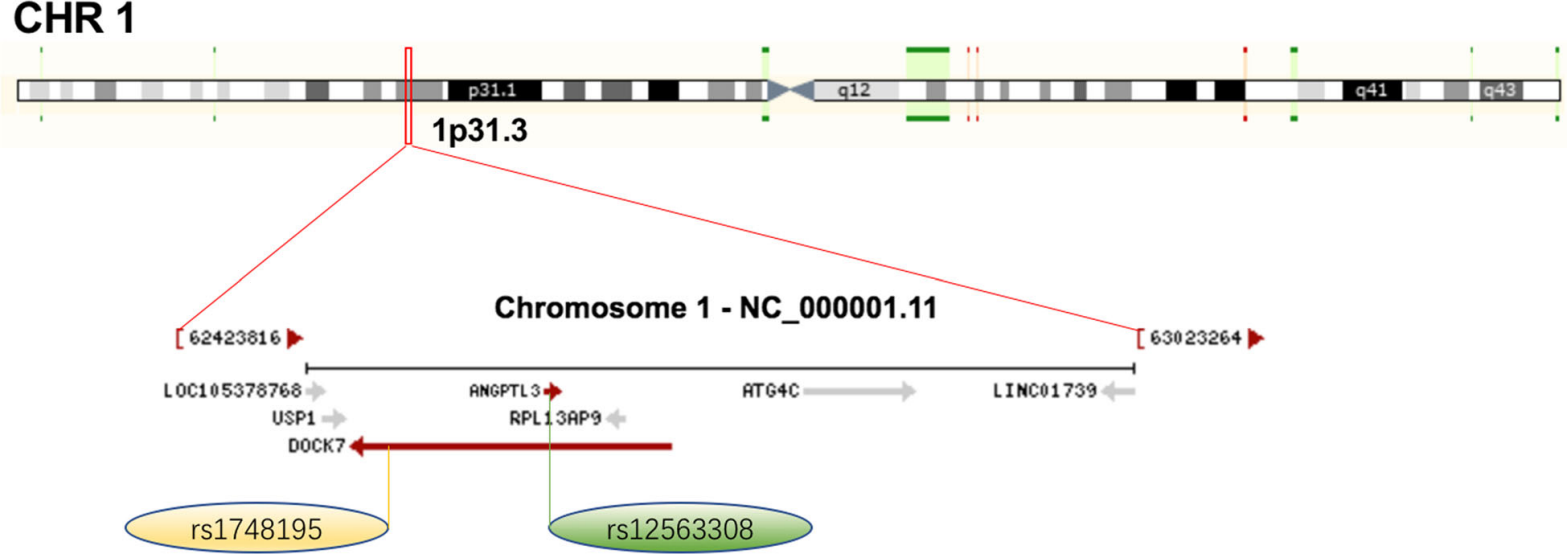
The positions of the *DOCK7* rs1748195 and *ANGPTL3* rs12563308 variants

**Fig. 4:**
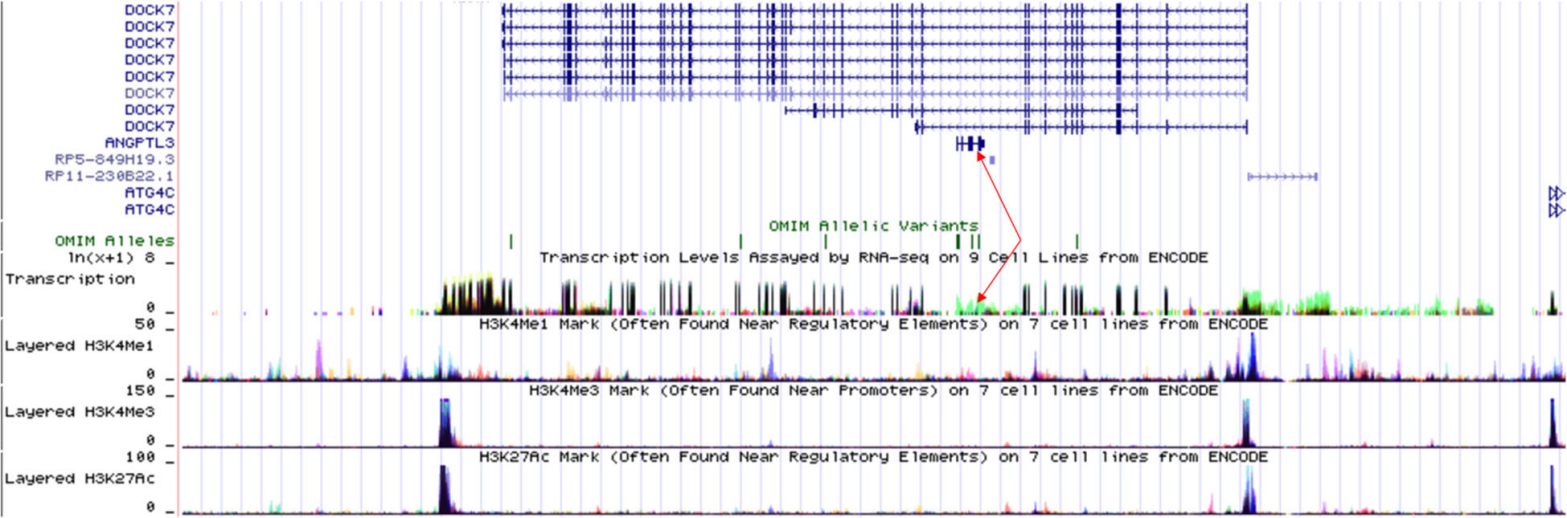
Annotation of genes in UCSC. The green peak was a part of transcription of the *DOCK7*, and the red arrows point to the *ANGPTL3* and transcription region

The association between the two SNPs and the risk of CHD and IS is not well known. In the current study, we showed that the *DOCK7* rs1748195 SNP and rs1748195G-rs12563308T haplotype were associated with an increased risk of CAD and the severity to coronary atherosclerosis, whereas the *ANGPTL3* rs12563308 SNP was associated with a decreased risk of CAD. The interactions of the two SNPs and several environmental factors on serum lipid levels, the severity to coronary atherosclerosis, and the risk of CAD and IS were also detected. The reasons for these differences may be related to the following factors: 1) The *DOCK7* rs1748195 SNP is allele C > G conversion, whereas the *ANGPTL3* rs12563308 SNP is T > C conversion. The G/T allele carriers had higher risk of CAD than the G/T allele non-carriers (CC genotypes). 2) There may be different linkage-disequilibrium of the two SNPs and/or carry different haplotypes with other SNPs. 3) Although the *ANGPTL3* lies within an intron of *DOCK7* (Fig. 3), the expression products after mutation may be different. Study has found that TATA-box binding protein associated factor 1 (TAF1) as an enhancer for transcription factor binding with *ANGPTL3* to regulate the *DOCK7* [33]*.* Thus, the *ANGPTL3* can influence the transcription of the *DOCK7* (Fig. 4)*.* 4) In addition, CAD and IS are two different diseases. Although a strong association has been found between CAD and IS, there are also many different aspects between the two diseases. The CAD and IS cases in this study were independent individuals who were exposed to different genetic and environmental risk factors. The results of exposure to different lifestyle and environmental factors probably further modify the association of genetic polymorphisms and the risk of CAD and IS in our study populations. Two recent studies indicated that ANGPTL3 might be a promising therapeutic target in patients with dyslipidemia who are at risk of atherosclerotic cardiovascular disease [18, 34]. Dewey et al. [34] reported that a loss-of-function variant in *ANGPTL3* was associated with a 41% lower risk of CAD (OR = 0.59, 95% CI = 0.14-0.85, *P* = 0.004). They also found that the human anti-ANGPTL3 monoclonal antibody, evinacumab could cause a reduction in fasting TG levels of up to 76% and LDL-C levels of up to 23% in 83 healthy human volunteers. Stitziel et al. [35] also reported that *ANGPTL3* deficiency was associated with protection from CAD.

### Limitations

Several potential limitations should be acknowledged in this study. Firstly, the sample size was relatively small compared to many GWASes and replication investigations. Therefore, larger sample sizes are needed to confirm our findings. Secondly, although several confounders have been adjusted for the statistical analyses in this study, several environmental factors such as BMI and blood pressure were different between cases and controls. We could not completely exclude the potential influence of these factors on the results. Thirdly, we could not analyze the association of the *DOCK7-ANGPTL3* rs1748195 and rs12563308 SNPs and serum lipid levels in the CAD/IS patients because of the influence of lipid-lower drugs. Finally, it is well known that both CAD and IS are complex multifactorial disorder. Although we have detected the association of two *DOCK7-ANGPTL3* SNPs and their haplotypes with serum lipid levels and the risk of CAD and IS in this study, there are still many unmeasured genetic and environmental factors and their interactions. Thus, our results still need to be confirmed in the other populations with larger sample sizes.

## Conclusions

This study showed that the *DOCK7* rs1748195 SNP and rs1748195G-rs12563308T haplotype were associated with an increased risk of CAD and the severity to coronary atherosclerosis, whereas the *ANGPTL3* rs12563308 SNP was associated with a decreased risk of CAD. The interactions of the two SNPs and several environmental factors on serum lipid levels, the severity to coronary atherosclerosis, and the risk of CAD and IS were also observed. These results suggest that the detection of these *DOCK7-ANGPTL3* SNPs in our study population may be useful for early diagnosis and future individualized treatment of dyslipidemia, CAD and IS.

## Abbreviations

ANCOVA: Analysis of covariance
ANGPTL3: Angiopoietin like 3 gene
Apo: Apolipoprotein
BMI: Body mass index
CAD: Coronary artery disease
CAS: Coronary atherosclerosis
CI: Confidence interval
DNA: Deoxyribonucleic acid
DOCK7: Dedicator of cytokinesis 7 gene
GWASes: Genome-wide association studies
HDL-C: High-density lipoprotein cholesterol
HWE: Hardy-Weinberg equilibrium
IS: ISCHEMIC stroke
LD: Linkage disequilibrium
LDL-C: Low-density lipoprotein cholesterol
MAF: Minor allele frequency
non-HDL-C: Non-high density lipoprotein cholesterol
OR: Odds ratio
SNP: Single nucleotide polymorphism
TAF1: TATA-box binding protein associated factor 1
TC: Total cholesterol
TG: Triglyceride
VLDL: Very low density lipoprotein
